# The Digital Divide and Cognitive Disparities Among Older Adults: Community-Based Cohort Study in China

**DOI:** 10.2196/59684

**Published:** 2024-11-27

**Authors:** Yumeng Li, Chen Liu, Jiaqing Sun, Junying Zhang, Xin Li, Zhanjun Zhang

**Affiliations:** 1 State Key Laboratory of Cognitive Neuroscience and Learning Beijing Normal University Beijing China; 2 Department of Management London School of Economics and Political Science London United Kingdom; 3 Institute of Basic Research in Clinical Medicine China Academy of Chinese Medical Sciences Beijing China

**Keywords:** digital divide, internet use, cognitive aging, mild cognitive impairment, socioeconomic status, resource inequality, cognitive disparities, cognitive function, elderly, older adult, aging, community-based, cohort study, China, ANCOVA, mixed linear model, Cox proportional hazards, mental health, internet, digital health, information and communication technologies, ICT, mHealth

## Abstract

**Background:**

The widespread adoption of information and communication technologies (ICTs) further deepens disparities in resource access, particularly among the aging population. However, the relationship between these factors and their resulting impact on cognitive abilities remains uncertain.

**Objective:**

This study aims to investigate the potential impact of the digital divide on individuals’ cognitive function and its association with the development and reversion of mild cognitive impairment (MCI).

**Methods:**

This cohort study used data from Beijing Aging Brain Rejuvenation (BABRI) study applying a multistage cluster sampling design between 2008 and 2020. The digital divide was quantified by the frequency of using ICTs. Analysis of covariance (ANCOVA), mixed linear models, and Cox proportional hazards models were used to model the association of digital divide and multidomain cognition.

**Results:**

Among the 10098 participants, nearly half (n=4941, 48.9%) faced the digital divide, which was associated with a worse performance in processing speed (F_10096_=10.67; *P*<.001; effect size *r*=0.42), rather than memory, executive function, and language. The model indicated that individuals’ physical and mental health, combined with their educational and occupational prestige, influenced the resources they attained, which ultimately caused the digital divide. Moreover, longitudinal data revealed that older adults who successfully crossed the digital divide during the tracking process and those who had already done so prior to tracking showed significantly slower rates of decline in processing speed (B=–1.98, *P*<.05; B=–2.62, *P*<.01) and general cognitive function (B=3.50, *P*<.001; B=3.13, *P*<.01). Additionally, overcoming the digital divide was also associated with a lower risk of developing MCI (hazard ratio [HR] 0.5, 95% CI 0.34-0.74; HR 0.43, 95% CI 0.29-0.62) and a greater probability of reversion from MCI to normal cognition (HR 6, 95% CI 3.77-9.56; HR 9.22, 95% CI 5.63-15.11).

**Conclusions:**

Overcoming the digital divide was significantly associated with improved cognitive function, a slower aging rate in cognitive performance, a reduced risk of developing MCI, and a higher likelihood of reverting from MCI to normal cognition.

## Introduction

In today’s modern society, digital information and communication technologies (ICTs) have become indispensable in our daily lives. The “digital divide” has become a vivid metaphor to describe the perceived inequality of those who either are unable to make use of ICTs [1]. The digital divide has a profound impact on the older population(between 50 and 90 years), which is experiencing rapid growth in numerous countries, including China, where both absolute numbers and relative proportions are continuously increasing. This demographic has significantly lower rates of acceptance and utilization of ICTs, leading to an increasing trend of exclusion among older adults in the rapidly advancing information society [2,3].

This phenomenon can be better understood through the lens of the resources and appropriation theory of the digital divide [1,4,5]. The core hypothesis of this theory posits that disparities in personal characteristics (such as health and habits) and positional characteristics (such as education and occupation) contribute to unequal access to diverse resources (such as mental well-being and social relationships), thereby resulting in the digital divide. However, there is currently a scarcity of empirical studies that comprehensively incorporate various influential factors and investigate the relationship pathways underlying the digital divide.

The digital divide can potentially contribute to the adoption of a less engaging lifestyle throughout older adults’ lifespans, which may diminish their “cognitive reserve” (the resource of the brain can get to tolerate the age-related changes or pathology related to dementia) and ultimately hasten the deterioration of cognitive function [6]. Evidence suggests that daily internet use is associated with a reduced risk of mild cognitive impairment (MCI) [7]. Moreover, longitudinal research indicates that overcoming the digital divide is associated with a decline in the incidence of MCI among older adults after 10 years [8]. Nonetheless, the existing literature focuses too narrowly on cognitive domains and fails to consider the specificity of cognitive differences caused by the digital divide. Similarly, the longitudinal changes in multidomain cognitive function influenced by the digital divide have not been adequately considered. Additionally, while many studies have investigated the progression of MCI, some found that a considerable proportion of individuals with MCI, ranging from 24% to 50%, may revert to a normal cognition (NC) status [9,10]. Thus, it is crucial to consider how the digital divide influences both the development and reversion of MCI.

It can be foreseen that the digital divide will put the older population in a state of resource inequality, impacting their future cognitive development. Based on the large-scale Beijing Aging Brain Rejuvenation (BABRI) study database, this study will investigate the following aspects: (1) the potential relationship among various influential factors contributing to unequal resource distribution related to the digital divide; (2) the cross-sectional differences and longitudinal aging rate alternations in multidomain cognitive function caused by the digital divide; and (3) the associations between the digital divide and the development and reversion of MCI, while controlling for normal risk factors.

## Methods

### Study Design

The BABRI cohort study is based on the registry of a large community population in Beijing, which collects comprehensive information on aging and tracks changes in cognitive function over the years [11]. All the participants were aged 50 years and above at the time of baseline enrollment, could live independently, did not have nervous system diseases or psychiatric disorders, and had 6 or more years of formal education, which was required for the cognitive assessments. The study had a multistage cluster sampling design, and participants were mainly recruited from communities in Beijing between 2008 and 2019. A total of 10098 qualified participants were recruited from the communities of these districts. All participants registered in BABRI were visited every 2 or 3 years over the total duration of the 20-year project.

This study will exclude longitudinal participants in the next phase of analysis based on the following criteria: (1) clinical diagnoses of neurodegenerative diseases (eg, Alzheimer disease and Parkinson disease), serious brain diseases (eg, severe cerebrovascular diseases, brain tumors, and brain trauma) or psychiatric disorders (eg, severe major depression disease, bipolar disorder, and schizophrenia); (2) history of substance or alcohol abuse/dependence; and (3) missing assessment indicators for the digital divide during the tracking process.

### Measurements

The Resources and Appropriation Theory of the digital divide was developed over a 10-year period, culminating in its full and mature presentation [5]. The core hypothesis of this theory posits that individuals’ characteristics and status lead to unequal access to resources, ultimately contributing to the emergence of the digital divide. In this study, the influencing factors of the digital divide were classified into 3 main parts. The independent variables included personal and positional categories, while the mediation variable included the resources.

### Personal Categories

#### Overview

Personal categories encompass various factors, such as physical health, which includes subjective health status, BMI, and chronic disease; mental health, including the Geriatric Depression Scale (GDS) and Facial Affect Coding System (FACE); and other factors like smoking and alcohol consumption.

#### Physical Health: Subjective Health Status, BMI, and Chronic Diseases

Subjective health status refers to a question asking participants to evaluate their overall health status with the following options: “good,” “fair,” “poor,” or “very poor.” BMI is calculated by dividing a person’s weight in kilograms by their height in meters squared. The participants’ medical histories were collected regarding hypertension, diabetes, and hyperlipidemia. This information was validated using the diagnosis and management records from community health service institutions.

#### Mental Health

The GDS is a standardized depression scale for older adults [12]. The FACE scale was used to measure subjective well-being, with higher scores indicating lower levels of well-being among older adults.

#### Daily Unhealthy Habits

Smoke and alcohol consumption were binary variables indicating whether the participants smoked or consumed alcohol every day.

### Positional Categories

Positional categories included the variables reflecting participants’ social status, such as educational status, subjective social status, and occupational prestige. Educational status refers to an individual’s specific duration of education. Subjective social status was assessed through the question, “How would you evaluate your current socioeconomic status?” The responses were rated on a 4-point scale: rich, upper-middle, lower-middle, and poor. Occupational prestige was measured using the Occupational Prestige Scale based on data from the Chinese national survey [13].

### Resources

The resources included economic, mental, and social resources. Economic resources refer to an individual's current monthly income, categorized into 13 levels based on increments of 500. Mental and social resources were obtained through a weighted summation based on relevant items derived from the Leisure Activity Scale (LAS), which is a 5-point scale that consists of 23 subitems used to assess cognition and social activity engagements in daily life [14].

### Quantifying the Digital Divide

The quantification indicator of the digital divide includes an item from the LAS about the frequency of using ICT: “How often do you use computer and mobile phone?” [15-17]. Participants with scores of 0 (never), 1 (≥ once a year), and 2 (≥ once a month) were classified into the Digital Divide (DD) group, while those with scores of 4 (≥once a week) and 5 (every day) into the Overcoming the Digital Divide (ODD) group. The DD group indicated individuals facing the digital divide, while the ODD group represented those who overcame it.

For cross-sectional data, participants were classified only into DD and ODD groups. To investigate the impact of ODD on cognitive aging using longitudinal data, participants were categorized into 3 groups. Two of these groups represented participants who remained in the DD or ODD group, while the third group, known as the Transition (Trans) group, included participants who moved from the DD group to the ODD group during the tracking process. Participants who transitioned from the ODD to the DD group were excluded, as crossing the digital divide is considered a relatively stable state. A transition from ODD to DD may indicate uncontrollable external factors, which are not the focus of this study.

### Cognitive Assessments

All participants underwent a battery of neuropsychological tests at the baseline recruitment [11]. The assessment involved general cognitive ability and cognitive function across 5 domains: memory, language, attention, visuospatial abilities, and executive function. General cognitive ability was tested using the Chinese version of the Mini-Mental State Examination (MMSE). Memory was tested using the Auditory Verbal Learning Test (AVLT) and the Rey-Osterrich Complex Figure (ROCF) test [18]. Executive function was tested using the Stroop Color Word Test (SCWT) and the Trail Making Test (TMT) [19]. Spatial processing was assessed using the Clock Drawing Test (CDT) [20] and the Rey-Osterrieth Complex Figure-Copy (R-Ocopy) test [18]. Attention was evaluated using the Symbol Digit Modification Test (SDMT) [21] and the Trail Making Test Part A (TMT-A) [19]. Language was tested using the Boston Naming Test (BNT) and the Verbal Fluency Test (VFT).

### MCI Diagnostic Criteria

The diagnostic criteria for MCI included the following [22]: individuals had to be without dementia, exhibit subjective cognitive decline, and present with at least 1 cognitive domain displaying 2 test scores that fell below 1.5 SD from the mean score of the same age and education level group in objective tests. Additionally, their general cognitive abilities and daily functioning had to be typically unaffected.

### Demographic Variables

Demographic variables included age, gender marital status, and residential status. Marital status included 3 options: married, divorced, and widowed. Residential status involved 3 options: living alone, living with a spouse, and living with children.

### Statistical Analysis

The participants’ baseline characteristics were presented as mean (SD) for continuous variables, while categorical variables were represented as frequencies and proportions.

The Z refers to the Z value obtained from performing a Mann-Whitney test. Intergroup variations in influential factors between the DD and ODD groups were assessed through independent sample *t* tests, chi-square tests, and nonparametric tests. We also used Cohen *d* and Cramer *V* to calculate effect sizes [23,24].

An intermediary model was constructed to elucidate the impact mechanism of the digital divide, using personal and positional characteristics as independent variables, resource acquisition as a mediating variable, and the digital divide as the dependent variable.

Intergroup differences in cognitive abilities were examined using analysis of covariance (ANCOVA) controlling for age, education level, gender, and chronic diseases. Binary logistic regression was employed to investigate the predictive impact of the digital divide variable on the occurrence of MCI.

A linear Bayesian change-point regression was performed on the age group–averaged data to compare the decline trajectories of multidomain cognition among the older population.

To effectively compare the impact of cognitive abilities among the DD, Trans, and ODD groups, dummy variable coding was applied to the longitudinal data to incorporate the effects of Trans-DD and ODD-DD in the model.

The mixed linear model (MLM) was used to examine the influence of the digital divide variable on the rate of cognitive aging at an individual level. Initially, we established the null model and unconditional growth model. The null model was used to determine the hierarchical structure of the longitudinal data for different cognitive functions, which was suitable for MLM analysis. The unconditional growth model was used to identify significant aging patterns in various cognitive functions over time. After selecting these 2 models, we constructed the full model that encompassed the following: (1) Level 1, which described individual cognitive level aging patterns, and (2) Level 2, which investigated the influence of the digital divide variable on aging patterns across multiple cognitive abilities in individuals.



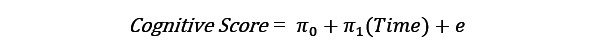





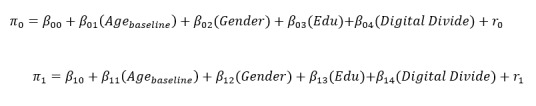



To investigate the impact of the digital divide variable on individual MCI development and outcomes, we employed the Cox proportional hazards model. Two models were developed, the first focusing on the progression from NC to MCI and the second focusing on the transition from MCI to NC.

### Ethical Considerations

The study was conducted in accordance with the institutional review board at the Imaging Center for Brain Research at Beijing Normal University (ICBIR_A_0041_002_02) and was approved in March 2015. We used STROBE (Strengthening the Reporting of Observational Studies in Epidemiology) as our reporting framework. Written informed consent followed by sociodemographic information was obtained from the participants before initiating the neuropsychological tests. All participants were reimbursed with daily necessities valued at 20 RMB (approximately $10 US) and provided with a free screening report covering multiple domains of cognition as a token of appreciation.

## Results

### Overview

This study included 10,098 participants in the cross-sectional analysis, with a mean age of 66.7 (SD 7.9) years. Among them, 6095 (60.4%) were female and 4003 (39.6%) were male. The mean educational level was 10.7 (SD 3.5) years), with 4941 (48.9%) in the DD group and 5157 (51.1%) in the ODD group. The longitudinal data analysis included 2092 participants, categorized into the DD, Trans, and ODD groups.

Among these participants, tracking outcomes fell into several categories: stable NC (NC→NC: N=1473), progression from NC to MCI (NC→MCI, N=190), stable MCI (MCI→MCI: N=193), and improvement from MCI to NC (MCI→NC, N=201).

### Characteristic Differences Among Older the Population Facing the Digital Divide

As shown in Table 1, the personal characteristics of the ODD group include hyperlipidemia, low depression scores, high subjective well-being, and nonsmoking. Their positional characteristics included higher education levels and higher self-perceived socioeconomic status. Additionally, the ODD group benefitted from greater access to economic, mental, and social resources.

**Table 1:** Characteristics and the digital divide among the older population.

Characteristics				DD^a^ (n=4941)		ODD^b^ (n=5157)		*t*/χ²/Z		FDR^c^-adjusted *P* value		Cohen *d*
**Demographic information**												
	Age, mean (SD)		67.65 (7.88)		64.60 (7.74)		19.66		<.001		0.39	
	Female, n (%)		2908 (58.9)		3187 (61.8)		9		.01		0.03	
	Married, n (%)		4060 (84.1)		4502 (89.6)		143.51		<.001		0.12	
	Divorced, n (%)		659 (13.6)		336 (6.7)		—^d^		—		—	
	Widowed, n (%)		109 (2.3)		185(3.7)		—		—		—	
	Live alone, n (%)		458 (10.8)		372 (8)		61.53		<.001		0.08	
	Live with spouse, n (%)		3170 (74.9)		3811(81.7)		—		—		—	
	Live with children, n (%)		605(14.3)		480 (10.3)		—		—		—	
**Personal characteristics**												
	**Physical health**											
		Subjective health, median (IQR)	1876.5 (2222.0)		1889.5 (2037.5)		–2.135		.05		0.04	
		BMI, mean (SD)	36.19 (820.14)		25.70 (120.96)		0.91		.36		—	
		Hypertension, n (%)	2342 (49.5)		2414 (48.6)		0.75		.39		0.01	
		Diabetes, n (%)	1024 (21.7)		4948 (21.6)		0.93		.93		0.001	
		Hyperlipidemia, n (%)	1328 (31.4)		1969 (42.5)		114.97		<.001		0.11	
	**Mental health**											
		SWB^e^, mean (SD)	2.63 (1.19)		2.51 (1.29)		4.25		<.001		0.1	
		GDS^f^, mean (SD)	8.18 (5.96)		7.61 (5.63)		4.93		.004		0.1	
	**Lifestyle**											
		Smoking, n (%)	1413 (29.6)		1242 (24.5)		31.74		<.001		0.06	
		Drinking, n (%)	1183 (27.8)		1320 (27.5)		0.18		.68		0.004	
	**Positional characteristics**											
		Education, mean (SD)	9.35 (3.43)		11.76 (3.16)		–36.73		<.001		0.73	
		Self-perceived status, median (IQR)	4451.5 (2450.0)		4926.5 (2363.5)		–7.36		<.001		0.15	
		Occupation, mean (SD)	0.61 (0.11)		0.61 (0.11)		0.84		.40		0	
**Resources**												
	Economic resources, median (IQR)		3828.5 (3437.5)		6268.0 (4087)		–36.66		<.001		0.78	
	Mental resources, mean (SD)		47.36 (18.66		72.09 (23.04)		–59.37		<.001		1.18	
	Social resources, mean (SD)		24.66 (13.49		35.83 (15.40)		–38.80		<.001		0.77	

^a^DD: Digital Divide group.

^b^ODD: Overcoming the Digital Divide group.

^c^FDR: false discovery rate.

^d^–: not applicable.

^e^SWB: subjective well-being

^f^GDS: Geriatric Depression Scale.

### Resources as Mediators in the Digital Divide

**Figure 1:**
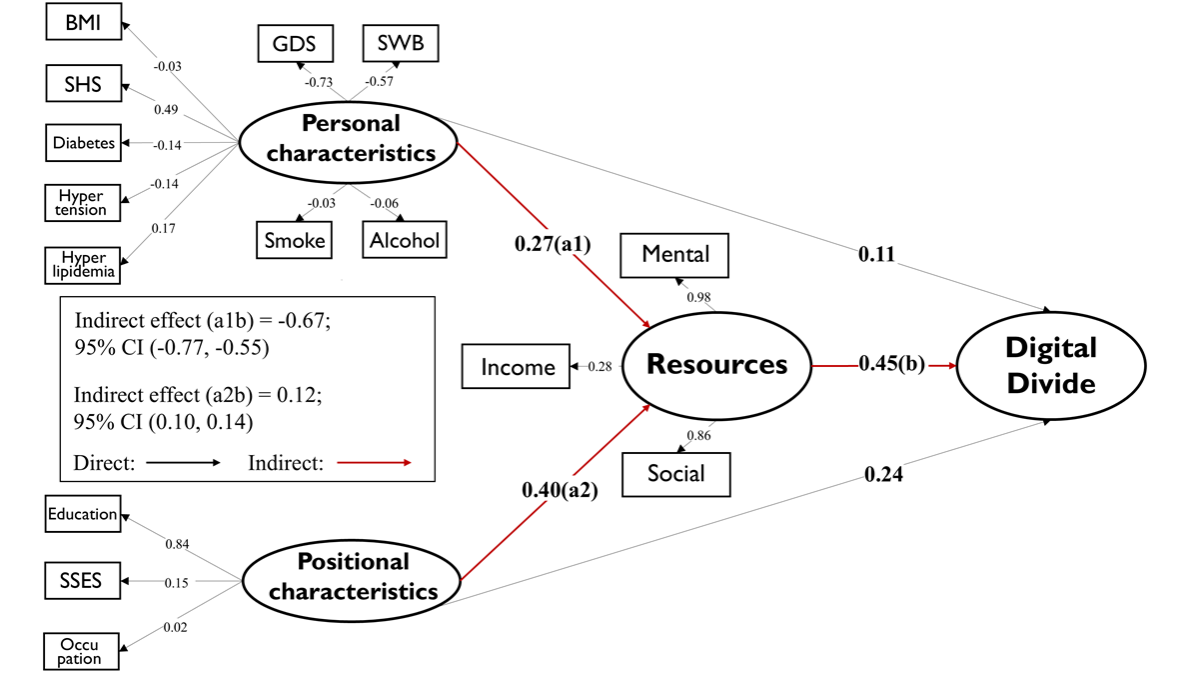
Mediation model Elucidating the Mechanism of the Digital Divide. The model is adjusted for the demographic variables (age, gender, marital status and residential status). Abbreviations: BMI: body mass index; SHS: subjective health score; GDS: Geriatric Depression Scale; SWB: subjective well-being; SSES: subjective socioeconomic scores.

The mediation model in Figure 1 illustrates the potential mechanisms behind the digital divide formation among older adults. It suggests that inequalities in individual characteristics and social status contribute to unequal access to economical, mental, and social resources, ultimately leading to the digital divide.

### Cognitive Differences Among Older Adults in the Digital Divide

Table 2 presents the differences in multidomain cognitive function between the DD and ODD groups. The results indicate that only the SDMT score of processing speed (F_10096_=10.67; *P*<.001) and working memory showed significant differences between the 2 groups. However, the effect size of processing speed was the largest (Cohen *d*=0.92).

**Table 2:** Digital divide and cognitive performance (corrected for age, sex, education, and chronic disease).

Cognitive domain			DD^a^ (n=4941), mean (SD)		ODD^b^ (n=5157), mean (SD)		*F* statistic		FDR^c^-adjusted *P* value		Cohen *d*
**General mental status**											
	MMSE^d^		26.12 (3.15)		27.51 (2.13)		1.44		.43		0.52
**Episodic memory**											
	AVLT^e^		23.48 (9.59)		27.75 (8.79)		0.12		.78		0.46
	R-Odelay^f^		10.52 (6.71)		13.70 (7.11)		2.15		.30		0.46
**Spatial processing**											
	CDT^g^		22.33 (5.87		24.29 (5.05)		0.97		.46		0.36
	R-Ocopy^h^		31.66 (6.63		33.96 (3.91)		0.48		.64		0.42
**Processing speed**											
	SDMT^i^		25.83 (10.98		35.92 (11.01)		10.67		<.001		0.92
	TMT-A^j^		74.33 (36.10		58.34 (22.93)		0.26		.69		0.53
**Executive function**											
	SCWT^k^	94.12 (35.84		79.81 (25.73)		1.02		.46		0.46	
	TMT-B^l^	205.4 (92.57		157.4 (62.20)		0.36		.66		0.61	
**Language**											
	VFT^m^	39.45 (9.57		45.48 (9.03)		0.01		.99		0.65	
	BNT^n^	20.86 (4.37		24 (3.13)		5.33		.07		0.83	
**Working memory**											
	DSTtotal^o^	11.91 (4.68)		12.65 (4.65)		7.93		0.03		0.16	

^a^DD: Digital Divide group.

^b^ODD: Overcoming the Digital Divide group.

^c^MMSE: Mini-Mental State Examination.

^d^FDR: false discovery rate.

^e^AVLT: Auditory Verbal Learning Test

^f^R-Odelay: Rey-Osterrieth Complex Figure Test delayed recall score.

^g^CDT: Clock Drawing Test.

^h^R-Ocopy: Rey-Osterrieth Complex Figure Test copy score.

^i^SDMT: Symbol Digit Modification Test.

^j^TMT-A: Trail-Making Test part A.

^k^SCWT: Stroop Color Word Test.

^l^TMT-B: Trail-Making Test part B.

^m^VFT: Verbal Fluency Test.

^n^BNT: Boston Naming Test.

^o^DSTtotal: Digital Span Test total score.

### Cross-sectional Association Between Overcoming the Digital Divide and Risk of MCI Incidence

The risk of developing MCI was significantly higher in the DD group compared to the ODD group (Table S1 in Multimedia Appendix 1), with an odds ratio (OR) of 3.06 (95% CI 2.74-3.42). Among other factors influencing MCI, such as age (OR 1.38, 95% CI 1.24-1.54), diabetes (OR 1.23, 95% CI 2.74-3.42], and hyperlipidemia (OR 0.74, 95% CI 0.66-0.83), DD has the highest OR value.

### Longitudinal Evidence of the Digital Divide’s Influence on Cognitive Aging

Significant interindividual variations were observed for all cognitive abilities in the null model, indicating the potential for constructing the subsequent mixed linear model (Table S2 in Multimedia Appendix 1). However, in the unconditional growth model, the age-related change trend of CDT did not reach statistical significance, which prevented the construction of the full model.

Table 3 shows that the digital divide significantly influenced the aging rate of multidomain cognitive function. Compared to the ODD and Trans groups, the DD group exhibited a faster cognitive aging rate, including MMSE (B_13_=3.50, *P*<.001; B_14_=3.13, *P*=.002), processing speed (TMTA: B_13_=–1.98, *P*=.04; B_14_ =–2.62, *P*<.001), and language (BNT: B_13_=3.16, *P*=.002).

**Table 3:** Longitudinal evidence of the digital divide’s influence on cognitive aging^a^.

Category		Aging rate (B1j)					Initial state (B0j)					
		Age (B_11_)	Gender (B_11_)	Edu^b^ (B_12_)	Trans-DD^c^ (B_13_)	ODD-DD^d^ (B_14_)		Age (B_01_)	Gender (B_02_)	Edu (B_03_)	Trans-DD(B_03_)	ODD-DD (B_04_)
**General mental status**												
	MMSE^e^	–5.92^***^	–1.41	1.73	3.50^***^	3.13^**^		–6.63^***^	0.90	1.90	–1.95	–1.11
**Episodic memory**												
	AVLT^f^	–4.74^***^	–1.88	1.07	0.27	–0.30		1.07	–1.12	2.79^**^	1.18	2.13^*^
	RO-delay^g^	–3.15^**^	–1.79	1.57	1.94	1.47		–2.41^*^	3.83^***^	1.85	0.05	1.30
**Spatial processing**												
	RO-copy^h^	–2.66^**^	0.18	2.25^*^	1.44	0.72		1.42	0.27	1.44	0.32	1.36
**Processing speed**												
	SDMT^i^	–2.48^*^	2.39^*^	0.91	1.69	1.61		–3.54^***^	–4.07^***^	5.29^***^	2.44^*^	1.05
	TMT-A^j^	3.71^***^	–0.33	–1.91	–1.98^*^	–2.62^**^		–3.90^***^	–0.98	–2.821^*^	0.21	–0.26
**Executive function**												
	StroopC^k^	3.42^**^	0.95	0.34	-0.94	–0.84		–1.63	1.80	–2.04^*^	–0.13	–1.04
	TMT-B^l^	4.74^***^	–0.18	–2.28^*^	–1.23	–1.71		–3.06^**^	–0.56	–2.80^**^	–1.13	–2.11^*^
**Working Memory**												
	DST^m^	–4.16^***^	–2.02^*^	1.31	-0.84	–1.42		0.51	2.66^**^	3.77^***^	2.73^**^	4.94^***^
**Language**												
	VFT^n^	–5.61^***^	–0.67	0.93	1.61	1.15		5.58^***^	0.07	2.78	0.66	2.20^*^
	BNT^o^	–3.83^***^	–4.78^***^	2.08^*^	3.16^**^	1.34		0.73	8.10^***^	2.67^**^	–0.94	2.09^*^

^a^The coefficients of MLM (B1j, B0j) refer to the function of Level 1 and Level 2 (see the Statistical Analysis subsection for more information).

^b^Edu: education level.

^c^Trans-DD: participants who overcame the digital divide only during the tracking process compared to participants who remained facing the digital divide.

^d^ODD-DD: participants who consistently overcame the digital divide throughout the entire tracking process compared to those who remained facing the digital divide.

^e^MMSE: Mini-Mental State Examination.

^f^AVLT: Auditory Verbal Learning Test

^g^RO-delay: Rey-Osterrieth Complex Figure Test delayed recall score.

^h^R-Ocopy: Rey-Osterrieth Complex Figure Test copy score.

^i^SDMT: Symbol Digit Modification Test.

^j^TMT-A: Trail-Making Test part A.

^k^StroopC: Stroop Color test.

^l^TMT-B: Trail-Making Test part B.

^m^DST: Digital Span Test.

^n^VFT: Verbal Fluency Test.

^o^BNT: Boston Naming Test.

***P<.001; **P<.01; * *P*<.05.

### Longitudinal Evidence of the Digital Divide’s Influence on the Development and Reversion of MCI

As Table 4 shows, compared to the DD group, both the Trans and ODD groups had a significantly lower probability of developing MCI (Trans-DD: hazard ratio [HR] 0.50, 95% CI 0.34-0.74; ODD-DD: HR 0.43, 95% CI 0.29-0.62), while they have a significantly greater probability of reversion from MCI to the healthy (Trans-DD: HR 6, 95% CI, 3.77-9.56; ODD-DD: HR, 9.22, 95% CI, 5.63-15.11).

**Table 4:** Longitudinal evidence of the digital divide’s influence on the development and reversion of MCI^a^.

Independent variables	MCI development (NC^b^→MCI) (n=190), HR^c^ (95% CI)	MCI reversion (MCI→NC) (n=201), HR (95% CI)
trans-DD^d^	0.5 (0.34-0.74)	6 (3.77-9.56)
ODD-DD^e^	0.43 (0.29-0.62)	9.22 (5.63-15.11)
Gender	0.76 (0.57-1.02)	1.06 (0.78-1.45)
Age	1.08 (1.06-1.10)	1.01 (0.99-1.03)
Edu^f^	0.93 (0.90-0.97)	0.99 (0.95-1.04)
Hypertension	1.36 (1.01-1.82)	0.97 (0.72-1.31)
Diabetes	1.43 (1.03-1.98)	1.2 (0.89-1.63)
Hyperlipidemia	1.05 (0.79-1.99)	0.88 (0.67-1.17)

^a^MCI, mild cognitive impairment.

^b^NC: normal cognition.

^c^HR: hazard ratio.

^d^Trans-DD: participants who overcame the digital divide only during the tracking process compared to participants who remained facing the digital divide.

^e^ODD-DD: participants who consistently overcame the digital divide throughout the entire tracking process compared to those who consistently remained facing the digital divide.

^f^Edu: education level.

### Sensitivity Analyses

To verify that the cross-group differences between the DD group and the ODD group were not due to differences in age and education level, we randomly selected 50 participants from the DD group. Using propensity score matching, we matched these participants with those from the ODD group based on age, education level, and gender. We conducted the same statistical analysis using identical procedures, and the results were consistent, largely replicating the initial findings (Tables S3-S5 in Multimedia Appendix 1).

## Discussion

### Principal Findings

This population-based cohort study verified the relationship pathway of the digital divide driven by resource inequality influenced by various factors. It also revealed a significant association between the digital divide and both cross-sectional and longitudinal differences in cognition, as well as the development and reversion of MCI. The population that came the digital divide exhibited higher scores in processing speed compared to those who suffered from the digital divide. Additionally, overcoming the digital divide was associated with a slower aging rate in MMSE, processing speed, and language skills, along with a reduced probability of developing MCI and an increased probability of transitioning from MCI into a healthy state. This study examines, for the first time, the impact of the digital divide on multiple cognitive domains and the reversion of MCI in the aging population. Additionally, a model of the digital divide among older adults was developed. Due to its correlation with the use of social media, previous studies have often associated the digital divide with mental health issues [15,25]. Nonetheless, our work aims to shift public attention to the cognitive abilities essential for supporting the daily functioning of the aging population, in response to the potential disability that may arise during their aging process.

As our results have demonstrated, the digital divide is significantly associated with the development and reversion of MCI. Given the numerous clinical treatment failures observed in Alzheimer disease, MCI has increasingly been recognized as a crucial opportunity for disease treatment and intervention [26]. Our study’s innovative findings clearly indicate that crossing the digital divide plays a significant role in the conversion from MCI to NC. Despite a tracking period of only 2 to 3 years, the conversion rate was more than 6 times higher in the DD group. This provides valuable insights for early intervention in the MCI stage of Alzheimer disease, as the utilization of ICTs is cost-effective and has a significant impact on neuroplasticity. The underlying reason for its considerable benefits is that ICTs reshape individuals’ interactive environments, exposing users to a wealth of environmental stimuli and information [27,28]. This is especially applicable to older adults because bridging the digital divide exposes them to a new lifestyle characterized by abundant information, necessitating a learning process.

Consistent with previous studies on the purpose of online cognitive training to improve cognitive function in older adults, our findings provide further epidemiological evidence for neural plasticity and cognitive improvement during the aging process. We recommend that cognitive training methods be developed and integrated with digital platforms and portable devices to delay age-related cognitive decline. We also recommend exploring novel approaches for early intervention in pathological aging.

Additionally, our findings indicate that the impact of the digital divide on different cognitive functions is selective. Overcoming the digital divide, both at the cross-sectional group level and the longitudinal developmental level, has a significant positive effect on processing speed. The continuous influx of online information and multimedia streams with multiple presentation modes during the use of ICTs encourages older adults to engage in attention-switching and simultaneous attention to multiple tasks, thereby enhancing the processing speed of cognitive resources [29]. Furthermore, studies have shown that many cognitive-related variables are considered to reflect processing speed, especially in the aging process [30]. Therefore, the influence of the digital divide on processing speed is also evident in overall cognitive abilities (MMSE), according to our findings.

### Limitations

This study has a few limitations. First, during the third wave of longitudinal data collection, the sample size of the data was lower than expected due to the COVID-19 pandemic. Second, relatively few variables were used to measure mental health, which may not fully capture the psychological factors affecting the participants. Third, regarding the quantification of the digital divide, this study did not distinguish the specific uses of ICTs. It is possible that some participants passively received phone calls and messages without actively engaging with ICTs to receive more stimuli. They may have been misclassified and placed into the ODD group.

### Conclusion

In this cohort study, the underlying mechanism of the digital divide among older adults was influenced by their personal physiological and psychological characteristics, as well as their positional status, impacting their access to economic, cognitive, and social resources. These factors ultimately determined their ability to overcome the digital divide. Based on the cross-sectional association, processing speed shows the strongest effect size in relation to the digital divide. Furthermore, overcoming the digital divide could delay the decline of general cognition, processing speed, and certain language functions. It also decreases the development of MCI and increases the likelihood of reversion from MCI. These findings can help inform strategies for dementia prevention and cognitive reserve strengthening in later life, particularly in the context of modifiable daily routines.

## Appendix

Multimedia Appendix 1 Supplementary materials.

## Abbreviations

ANCOVA: analysis of covariance
AVLT: Auditory Verbal Learning Test
BABRI: Beijing Aging Brain Rejuvenation
BNT: Boston Naming Test
CDT: Clock Drawing Test
DD: Digital Divide (participant group)
FACE: Facial Affect Coding System
GDS: Geriatric Depression Scale
LAS: Leisure Activity Scale
MCI: mild cognitive impairment
MLM: mixed linear model
MMSE: Mini-Mental State Examination
NC: normal cognition
ODD: Overcoming the Digital Divide (participant group)
OR: odds ratio
ROCF: Rey-Osterrieth Complex Figure test
R-Ocopy: Rey-Osterrieth Complex Figure-Copy test
SCWT: Stroop Color Word Test
SDMT: Symbol Digit Modification Test
STROBE: Strengthening the Reporting of Observational Studies in Epidemiology
TMT: Trail Making Test
TMT_A: Trail Making Test Part A
Trans: Transition (participant group)
VFT: Verbal Fluency Test

## Data Availability

The data sets analyzed during the study are available from the corresponding author upon reasonable request.
