# Supplementary material for: The Digital Divide and Cognitive Disparities Among Older Adults: Community-Based Cohort Study in China
Source: J Med Internet Res. 2024 Nov 27;26:e59684. doi: 10.2196/59684 (PMC11635332; doi:10.2196/59684)
Supplement: Multimedia Appendix 1 [file jmir_v26i1e59684_app1.docx]

**Supplementary Materials**

**Supplemental Table 1. The cross-sectional association of overcoming Digital Divide and risk of incidence of MCI**

|  | **OR** | **95%CI** | ***P*** |
| --- | --- | --- | --- |
| **Digital Divide** |  |  |  |
| ODD | 1 | (2.74 - 3.42) | <.001 |
| DD | 3.06 |  |  |
| **Age** |  |  |  |
| < 65 year | 1 | (1.24 - 1.54) | <.001 |
| > 65 year | 1.38 |  |  |
| **EDU** |  |  |  |
| College degree | 1 | (1.08 – 2.12) | 0.017 |
| Non-college | 1.51 |  |  |
| **Hyperlipidemia** |  |  |  |
| No | 1 | (0.66 – 0.83) | <.001 |
| Yes | 0.74 |  |  |
| **Diabetes** |  |  |  |
| No | 1 | (1.08 – 1.40) | 0.002 |
| Yes | 1.23 |  |  |
| **Hypertension** |  |  |  |
| No |  |  | 0.079 |
| Yes |  |  |  |

Notes: The Hypertension was excluded from the whole model due to a lack of statistical significance in influencing the target event

**Supplemental Table 2. The cross-sectional association of overcoming Digital Divide and risk of incidence of MCI**

|  |  | **The Null Model** | | **The unconditional growth model** |
| --- | --- | --- | --- | --- |
|  |  | **Wihin-Variances** | **Between-Variances** | **Aging rate(π_1_)** |
| *General mental* | **MMSE** | 2.93^***^ | 630.12^***^ | -13.39^***^ |
| *status* |  |  |  |  |
| *Episodic memory* | **N1N5** | 29.54^***^ | 150.77^***^ | -14.30^***^ |
|  | **RO-delay** | 21.01^***^ | 95.96^***^ | -4.23^***^ |
| *Spatial processing* | **RO-copy** | 10.89^***^ | 423.15^***^ | -4.59^***^ |
|  | **CDT** | 21.74^***^ | 284.28^***^ | 1.72 |
| *Mental speed* | **SDMT** | 23.13^***^ | 144.95^***^ | -16.01^***^ |
|  | **TMTA** | 9.18 ^***^ | 125.51 ^***^ | 18.30^***^ |
| *Executive function* | **StroopC** | 6.42^***^ | 160.55^***^ | 18.05^***^ |
|  | **TMTB** | 14.89^***^ | 116.00^***^ | 21.58^***^ |
| *Working Memory* | **DST** | 21.73^***^ | 271.96^***^ | -4.42^***^ |
| *Language* | **VFT** | 26.28^***^ | 245.89^***^ | 12.29^***^ |
|  | **BNT** | 24.80^***^ | 366.14^***^ | -4.86^***^ |

The null model was utilized to determine the hierarchical structure of the longitudinal data for different cognitive functions, which was suitable for MLM analysis. The results indicated that the whin-level variability was significant in all domains of cognitive function, suggesting the feasibility of constructing subsequent models.

The unconditional growth model was used to identify significant aging patterns in various cognitive functions over time. The results indicated that most of the cognitive function declined with aging except the CDT. Thus, full models will be established for all cognitive abilities except for CDT.

**Supplemental Table 3. Characteristics and Digital Divide among older population (After PSM)**

|  | DD Group | ODD Group | t/χ²/Z | P_FDR | Cohen‘d |
| --- | --- | --- | --- | --- | --- |
|  | n = 2197 | n = 2197 |  |  |  |
| **Demographic information** |  |  |  |  |  |
| Age, mean ±SD | 67.04±7.80 | 66.92±7.52 | -0.56 | 0.58 | 0.39 |
| Female, n (%) | 1269(57.8%) | 1304(59.4%) | 1.15 | 0.28 | 0.02 |
| Married n(%) | 1824(85.0%) | 1899(88.7%) | 60.59 | <0.001 | 0.12 |
| Divorced, n(%) | 281(13.1%) | 150(7.0%) |  |  |  |
| Widowed, n(%) | 42(2.0%) | 93(4.3%) |  |  |  |
| Live alone, n(%) | 198(10.5%) | 179(9.1%) | 8.56 | 0.01 | 0.05 |
| Live with spouse, n(%) | 1429(76.0%) | 1577(52.5%) |  |  |  |
| Live with children, n(%) | 253(13.5%) | 218(11.0%) |  |  |  |
| **Personal characteristics** |  |  |  |  |  |
| **Physical Health** |  |  |  |  |  |
| Subjective health, mean rank | 2153.91 | 2152.08 | -0.053 | 0.96 | 0.04 |
| BMI, mean ±SD | 28.26±184.36 | 25.67±93.44 | 0.59 | 0.56 |  |
| Hypertension, n (%) | 1036(49.1%) | 1070(49.7%) | 0.13 | 0.72 | 0.01 |
| Diabetes, n (%) | 472(22.5%) | 483(22.5%) | 0.001 | 0.974 | 0 |
| Hyperlipidemia , n (%) | 609(32.4%) | 824(40.9%) | 30.35 | <0.001 | 0.09 |
| **Mental Health** |  |  |  |  |  |
| SWB, mean ±SD | 2.63±1.28 | 2.54±1.20 | 2.45 | 0.014 | 0.1 |
| GDS, mean ±SD | 7.61±5.63 | 7.18±5.96 | 3.42 | 0.001 | 0.1 |
| **LifeStyle** | 1024(21.7%) | 4948(32.6%) | 0.93 | 0.925 |  |
| Smoking, n (%) | 629(29.5%) | 532(24.7%) | 12.32 | <0.001 | 0.05 |
| Drinking, n (%) | 536(28.2%) | 545(26.7%) | 1.13 | 0.29 | 0.02 |
| **Positional characteristics** |  |  |  |  |  |
| Education, mean ±SD | 9.89±3.21 | 10.05±3.00 | 1.75 | 0.1 | 0.73 |
| Self-perceived status, mean rank | 2006.73 | 1967.38 | -1.25 | 0.21 | 0.15 |
| Occupation, mean ±SD | 0.61±0.12 | 0.61±0.11 | 0.8 | 0.42 | 0 |
| **Resources** |  |  |  |  |  |
| Economic Resources, mean rank | 1733.86 | 2343.43 | -16.64 | <0.001 | 0.78 |
| Mental Resources, mean ±SD | 48.10±18.71 | 70.69±23.15 | -59.37 | <0.001 | 1.18 |
| Social Resources, mean ±SD | 25.19±13.80 | 35.12±15.82 | -38.8 | <0.001 | 0.77 |

Abbreviations: BMI, body mass index; GDS, scores of the geriatric depression scale; M=Mean, *SD*=Standard deviation; DD, participants who failed to overcome the digital divide; ODD, participants who overcome the digital divide.

**Supplemental Table 4. ANCOVA: digital divide and the cognitive performance (After PSM)**

|  | **DD** | **ODD** | **F/χ²** | ***P_FDR*** |
| --- | --- | --- | --- | --- |
|  | **n = 2197** | **n = 2197** |  |  |
| MMSE | 26.36±3.00 | 27.23±2.33 | 2.24 | 0.13 |
| N1-N5 | 24.12±9.58 | 26.37±8.98 | 0.12 | 0.73 |
| RO-delay | 10.75±6.63 | 12.81±6.94 | 2.06 | 0.15 |
| CDT | 22.53±5.78 | 23.94±5.20 | 0.53 | 0.47 |
| RO-copy | 31.99±6.34 | 33.46±4.54 | 0.69 | 0.41 |
| SDMT | 27.02±10.72 | 32.70±10.46 | 3.97 | 0.04 |
| TMT-A | 71.70±33.91 | 62.09±24.61 | 1.55 | 0.21 |
| StroopCTime | 91.99±33.97 | 83.46±26.60 | 2.46 | 0.12 |
| TMT-B | 198.53±86.51 | 171.77±69.48 | 1.09 | 0.3 |
| VFT | 39.95±9.39 | 44.05±8.80 | 2.52 | 0.11 |
| BNT | 21.15±4.18 | 23.41±3.42 | 0.39 | 0.53 |
| DSTtotal | 11.22±2.19 | 12.23±4.47 | 1.69 | 0.19 |

**Notes: Corrected for age, sex, education and chronic disease**

Abbreviations: MMSE, Mini-Mental State Examination; AVLT, Auditory Verbal Learning Test; ROCF, Rey-Osterrich Complex Figure Test; SCWT, Stroop Color Word Test; TMT, Trail Making Test; CDT, Clock Drawing Test; SDMT, Symbol Digit Modification Test; BNT, Boston Naming Test; VFT, Verbal Fluency Test; DST, Digital Span Test. DD, participants who failed to overcome the digital divide; ODD, participants who overcome the digital divide.

**Supplemental Table 5. The cross-sectional association of overcoming Digital Divide and risk of incidence of MCI (After PSM)**

|  | **OR** | **95%CI** | ***P*** |
| --- | --- | --- | --- |
| **Digital Divide** |  |  |  |
| ODD | 1 | (1.82 - 2.50) | <.001 |
| DD | 2.14 |  |  |
| **Age** |  |  |  |
| < 65 year | 1 | (1.23 - 1.69) | <.001 |
| > 65 year | 1.44 |  |  |
| **EDU** |  |  |  |
| College degree | 1 | (1.48 – 2.10) | <.001 |
| Non-college | 1.76 |  |  |
| **Hyperlipidemia** |  |  |  |
| No | 1 | (0.62 – 0.88) | <.001 |
| Yes | 0.74 |  |  |
| **Diabetes** |  |  |  |
| No | 1 | (1.01 – 1.48) | 0.04 |
| Yes | 1.22 |  |  |
| **Hypertension** |  |  |  |
| No | 0.99 | (0.85-1.17) | 0.94 |
| Yes |  |  |  |
